# An experimental quantum Bernoulli factory

**DOI:** 10.1126/sciadv.aau6668

**Published:** 2019-01-25

**Authors:** Raj B. Patel, Terry Rudolph, Geoff J. Pryde

**Affiliations:** 1 Centre for Quantum Computation and Communication Technology and Centre for Quantum Dynamics, Griffith University, Brisbane 4111, Australia.; 2 Department of Physics, Imperial College London, Prince Consort Road, London SW7 2AZ, UK.

## Abstract

There has been a concerted effort to identify problems computable with quantum technology, which are intractable with classical technology or require far fewer resources to compute. Recently, randomness processing in a Bernoulli factory has been identified as one such task. Here, we report two quantum photonic implementations of a Bernoulli factory, one using quantum coherence and single-qubit measurements and the other one using quantum coherence and entangling measurements of two qubits. We show that the former consumes three orders of magnitude fewer resources than the best-known classical method, while entanglement offers a further fivefold reduction. These concepts may provide a means for quantum-enhanced performance in the simulation of stochastic processes and sampling tasks.

## Teaser

Quantum coherence and entanglement offer a quantum advantage, over classical physics, in a randomness processing task.

## INTRODUCTION

The pursuit of quantum computers (*1*, *2*) has uncovered a number of scenarios where quantum information processing offers a clear advantage over classical means. There exist certain tasks that are intractable using a classical computer but are made possible with quantum computing, supporting the notion of “quantum supremacy” (*3*). While there are examples where a quantum advantage may exist, unequivocal experimental proof is often unattainable.

A task of interest is that of randomness processing. An example is where a sequence of random variables, obeying a probability distribution, is transformed to produce a new random variable obeying a different probability distribution. Recently, this task has been identified by Dale *et al*. (*4*) as a basic primitive for which quantum information processing offers advantages over classical stochastic techniques. First, it was shown that the encoding of information on coherent quantum states gives access to a broader range of probability distributions that can be transformed. Second, their approach was shown to require fewer resources, where the resources are akin to the runtime of the task. This randomness processing task has widespread applicability across science and is rooted in processes that are typically simulated by Markov chain Monte Carlo methods. In addition, investigations in this area bear upon our fundamental understanding of quantum randomness (*5*). In particular, they offer a new avenue for understanding the difference between epistemological classical randomness, owing to noncontextual ignorance about the real state of a system, and quantum randomness, for which no such interpretation is possible.

Here, we present quantum photonic experiments where polarization qubits are used to encode sequences of random variables, which are governed by a probability distribution. From these random variables, algorithmic processing allows for construction of a new distribution while exhibiting a quantum advantage. We show that quantum coherence reduces the resource consumption, or runtime, by several orders of magnitude compared to the best-known classical method, while entanglement offers even further improvements. Before describing the details of our work, we set the scene with some simple examples illustrating the type of processing our work tackles.

Let us first consider the scenario where a fair coin $f(p)=\frac{1}{2}$ is to be simulated from a series of tosses of a classical coin with unknown bias *p*. Here, *p* is the probability of a heads outcome, and *p* ∈ (0, 1). Von Neumann’s solution (*6*) is to toss the coin twice, and if the outcomes are different output the value of the second coin toss, or repeat the procedure if they are the same. Now, suppose the task is to simulate the function *f*(*p*) = *p*^2^ for *p* ∈ [0, 1]. This can be achieved by tossing the coin twice. A head is outputted if each individual toss results in heads; otherwise, the output is tails. Some polynomials are well suited to this type of construction. While it is obvious that the function *f*(*p*) = 2*p*(1 − *p*) may be simulated by tossing a coin twice, the function *f*(*p*) = 3*p*(1 − *p*) requires noting that 3*p*(1 − *p*) = 3*p*^2^(1 − *p*) + 3*p*(1 − *p*)^2^ (*7*), and as such, the coin must be tossed three times.

**Fig. 1:**
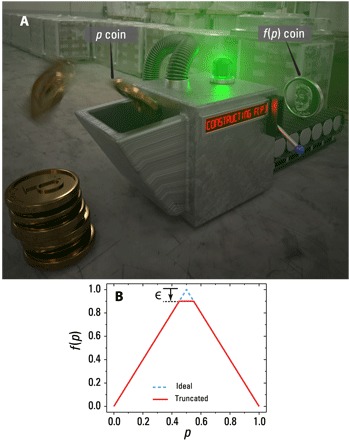
The classical Bernoulli factory. (**A**) Concept of sampling task. A sequence of iid coins, with an unknown bias *p*, are sampled and processed producing a new coin of bias *f*(*p*). (**B**) Doubling function *f*_^_(*p*) = 2*p*, as per Eq. 1. The dashed blue plot shows the ideal function, which cannot be constructed classically. The workaround is to truncate the function by ε, shown by the solid red line.

In these examples, we have described the scenario of the so-called “Bernoulli factory” (*7*–*16*), illustrated in Fig. 1A. Here, one draws from a sequence of independent and identically distributed (iid) Bernoulli random variables (coin tosses), i.e., ℙ(*X* = 0 ≡ Heads) = *p* and ℙ(*X* = 1 ≡ Tails) = 1 − *p*, for an unknown *p*. They then process the samples and output a new Bernoulli variable with the probability of obtaining heads *f*(*p*) : (*S* ⊆ [0, 1]) → [0, 1]. The goal is to not only transform from one probability distribution to another but also to do so with as few coin flips as possible. In the Bernoulli factory, the number of coin flips can be interpreted as the number of times a Bernoulli random variable is queried from a generator, which is proportional to the runtime of the task (*13*, *14*).

These ideas were developed by Keane and O’Brien (*8*) who derived the necessary and sufficient conditions under which a Bernoulli factory exists for *f*(*p*). These conditions are (i) *f*(*p*) must be continuous, (ii) *f*(*p*) must not approach a value of 0 or 1 exponentially quickly near *p* = 0 or 1, and (iii) *f*(*p*) ≠ 0 or 1 for *p* ∈ (0, 1).

## RESULTS

### Quantum Bernoulli factory for *f*(*p*) = 2*p*

Replacing the classical coin with a quantum coin or “quoin,” of the form $|p\rangle =\sqrt{p}|0\rangle +\sqrt{1-p}|1\rangle$ can yield some remarkable advantages (*4*). The extension to quoins enables algorithmic processing of coherent superpositions and entangled states, with a classical output. We will refer to this as the quantum Bernoulli factory (QBF) and the classical version as the CBF. One interesting feature of the QBF is that an advantage can be gained with quantum coherence alone. The conditions imposed by Keane and O’Brien are now relaxed in the quantum setting, allowing a larger class of functions to be constructed.

The function we choose to study, and perhaps the most important, is the “Bernoulli doubling” function

f^(p)=2p≡{2pp∈[0,1/2]2(1−p)p∈(1/2,1] (1)

since it serves as a primitive for other factories (*9*). That is, the ability to sample from this function allows any other analytical function to be constructed that is bounded at less than unity in (0, 1). Notice that this function cannot be constructed classically since *f*_^_(0.5) = 1 violates condition (iii). In the classical setting, the workaround is to truncate the function by ϵ, where 0 < ϵ < 1, such that *f*_^_(*p*) = 2*p* ≅ min (2*p*, 1 − ϵ) (*8*, *9*, *11*–*13*), as shown in Fig. 1B. From (*4*), the QBF for Eq. 1 can be realized by first rewriting the function as f^(p)=1−1−4p(1−p) and performing a series expansion

f^(p)=∑k=1kmax(2kk)1(2k−1)22k(4p(1−p))k=∑k=1kmaxqkgk (2)

Here, *k* > 0, *q*_*k*_ is independent of *p*, and *g*_*k*_ = (4*p*(1 − *p*))^*k*^. Typically, *k*_max_ = ∞; however, in realistic experimental scenarios, finite *k*_max_ values are considered. This representation allows us to reduce the problem to finding a construction for *g*_*k*_—or *k* consecutive heads outcomes of tossing a *g*_1_-coin—where a *g*_1_-coin is defined as a coin with a bias *g*_1_(*p*) = 4*p*(1 − *p*). The main task is thus to efficiently produce such a *g*_1_-coin. Performing a joint two-qubit measurement on two *p*-quoins |*p*〉 ⊗ |*p*〉 in the Bell basis, $\left\{|\psi^{\pm }\rangle =(|01\rangle \pm |10\rangle )/\sqrt{2},|\phi^{\pm }\rangle =(|00\rangle \pm |11\rangle )/\sqrt{2}\right\}$, we find that

$$\begin{array}{c} \mathbb{P}(\phi^{-}|(\psi^{+}\cup \phi^{-}))={\left(2p-1\right)}^{2},\\ \mathbb{P}(\psi^{+}|(\psi^{+}\cup \phi^{-}))=4p(1-p)=g_{1}(p),\\ \mathbb{P}(\phi^{-}|(\psi^{+}\cup \phi^{-}))+\mathbb{P}(\psi^{+}|(\psi^{+}\cup \phi^{-}))=1 \end{array}$$ (3)

where ℙ(ψ^+^|(ψ^+^ ∪ ϕ^−^)) is read as the probability of measuring |ψ^+^〉 given that we restrict ourselves to measuring states |ψ^+^〉 or |ϕ^−^〉. The algorithm runs by first generating an index *k* with a probability *q*_*k*_. A joint measurement, in a restricted Bell basis, is then performed on two *p*-quoins. If *k* consecutive |ψ^+^〉 outcomes are obtained, then the toss of an *f*_^_(*p*)-coin is heads; otherwise, if the outcome of the measurement is |ϕ^−^〉, then the output is tails.

### Two-qubit experimental QBF

**Fig. 2:**
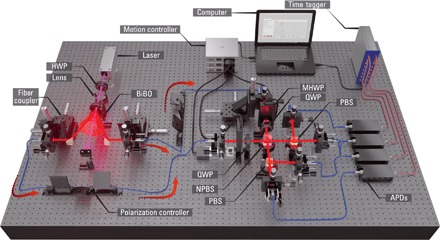
Experimental arrangement for the QBF using joint measurements of two *p*-quoins. A pair of H-polarized photons are generated via type I down-conversion in a nonlinear BiBO crystal. They are sent (indicated by red arrows) to a Bell-state analyzer arrangement containing additional MHWP, which set the bias value of each *p*-quoin, and QWPs, one at OA + 45° and one at OA − 45°, which enables |ψ^+^〉 and |ϕ^−^〉 to be identified. The photons interfere on a 50:50 NPBS, while the PBS enable H- and V-polarized photons to be separated spatially before being detected using single-photon APDs. Detection events are time-tagged and analyzed using a computer.

**Fig. 3:**
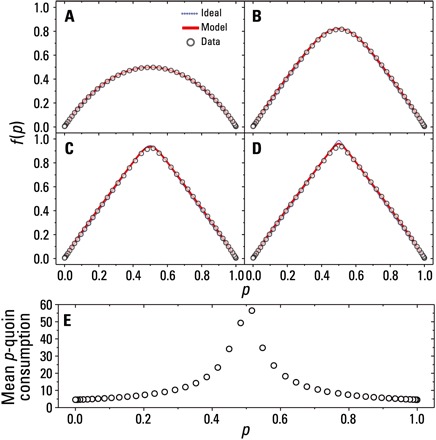
Experimental data for the two-qubit QBF. The function *f*_^_(*p*) = 2*p* is constructed using joint measurements of two *p*-quoins for (**A**) *k*_max_ = 1, (**B**) *k*_max_ = 10, (**C**) *k*_max_ = 100, and (**D**) *k*_max_ = 2000. The dotted blue lines are the ideal theoretical functions, and the red lines represent a model taking experimental imperfections into consideration. Error bars were too small to be included (see Materials and Methods). (**E**) Mean *p*-quoin consumption for *k*_max_ = 2000.

The required measurements are well suited to our linear optics implementation shown in Fig. 2. A 404 nm, vertically (V) polarized, continuous-wave laser beam pumps a nonlinear BiBO crystal generating a degenerate pair of horizontally (H) polarized photons, |*H*〉_1_ ⊗ |*H*〉_2_. The photons are spectrally filtered using longpass filters and 3 nm bandpass filters centered at 808 nm and are sent via single-mode fiber to a Bell-state analyzer. This particular arrangement contains additional motorized half-wave plates (MHWPs), which set the bias value of each *p*-quoin. It is well known that the standard linear optical Bell-state analyzer (*17*, *18*), relying on Hong-Ou-Mandel interference, is capable of unambiguously discriminating between the |ψ^+^〉 and |ψ^−^〉 Bell states. We implement an $X_{\frac{\pi }{2}}⊗X_{-\frac{\pi }{2}}$ operation on the qubits before the measurement using quarter-wave plates (QWPs) at optic axes (OA) ± 45°, which allows the desired states, |ψ^+^〉 and |ϕ^−^〉, to be identified. The photons interfere on a 50:50 nonpolarizing beamsplitter (NPBS), while polarizing beamsplitters (PBSs) enable H- and V-polarized photons to be separated spatially before being detected using single-photon avalanche diodes (APDs). The sequence of detection events is time-tagged, which allows us to identify the exact order in which a *g*_1_(*p*)-coin toss resulted in a heads (|ψ^+^〉) or a tails (|ϕ^−^〉). The data are postprocessed (see Materials and Methods for details) using a computer, and *f*_^_(*p*) is constructed. Figure 3 (A to D) shows the experimental data (circles) taken for *k*_max_ ∈ {1, 10, 100, 2000}. We see that the data agree strongly with the ideal theoretical plots (dotted lines). The red curves in each plot represent the expected data based on a model, which takes into account the nonideal splitting ratio of our NPBS, extinction ratios of our polarization optics, and any mode-mismatch in our interferometer. The experimentally measured Hong-Ou-Mandel two-photon interference visibility was found to be$(99.7_{-1.0}^{+0.3})\%$. The experimental data show an excellent agreement with our model. For lower values of *k*, the data show a more rounded peak near *p* = 0.5, which becomes sharper for larger *k*. In our experimental run, we were able to generate up to a single *g*_2036_(*p*) coin, i.e., up to 2036 consecutive heads outcomes of the *g*_1_(*p*) coin. Higher-order *g*_*k*_(*p*) coins are more susceptible to small experimental imperfections, which may lead to erroneous coincident detections. For more reliable statistics, and for comparison later on, in Fig. 3D, we restrict the expansion to *k*_max_ = 2000, where we obtain *f*_^_(0.5) = 0.935 ± 0.003. In Fig. 3E, we calculate the mean *p*-quoin consumption for each *f*_^_(*p*)-coin. Note that increasingly more quoins are required near *p* = 0.5, as we expect. We require an average (over *p*) of ≈ 11 quoins to construct *f*_^_(*p*) = 2*p* when using the quantum coherence and entangling measurements of two *p*-quoins.

### Single-qubit experimental QBF

**Fig. 4:**
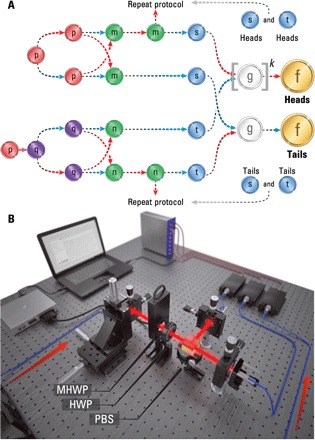
Construction of *f*_^_(*p*) = 2*p* using single-qubit measurements of *p*-quoins. (**A**) The algorithm we use (see the Supplementary Materials). The upper (lower) branch begins with the measurement of the *p*-quoin in the *Z*-basis (*X*-basis). Dashed red (blue) arrows indicate a heads (tails) outcome of the quoin toss. Failure to achieve the appropriate outcome requires the protocol to be repeated until success. (**B**) Experimental arrangement for the QBF using single-qubit measurements of *p*-quoins. Red arrows indicate photon inputs from the source (not shown). A single photon encounters a MHWP, which sets *p*. A HWP set to OA enables *Z*-basis measurements to be performed for each *p*. The partner photon, which serves as a herald, is detected directly by an APD. Setting the HWP to OA + 22.5 ° results in an *X*-basis measurement. Two sets of time-tag data are recorded, allowing *p* and *q*-quoins to be sampled.

We now show how *f*_^_(*p*) can be constructed using single-qubit measurements, where we exploit quantum coherence alone. To do so, we use the best-known algorithm for constructing *g*_1_(*p*) with single-qubit measurements, which was recently demonstrated using superconducting qubits (*19*). The algorithm makes use of additional, intermediate quoins denoted by *q*, *m*, *n*, *s*, and *t*, each with a unique probability distribution, which are derived from *p*-quoins. Figure 4A illustrates the procedure where red (blue) arrows indicate a heads (tails) outcome of a particular toss. A more thorough description is provided in the Supplementary Materials. To begin with, two *p*-quoins are produced, the second of which is measured in the *X*-basis to produce a *q*-quoin (lower branch). In the upper branch, the *p*-quoin is tossed twice, and if the outcome is different each time an *m*-quoin is produced with the outcome heads, otherwise, tails is outputted. Similarly, in the lower branch where a *q*-quoin is tossed twice with different outcomes, an *n*-quoin with a heads outcome results. The *m*- and *n*-quoins are both tossed twice. In each case, if the first toss results in tails, a new quoin is produced, *s* or *t*, with a tails outcome. If, however, the first toss gives heads and the second gives tails, then heads is outputted in each case. Otherwise, the protocol is repeated from the beginning, and two *p*-quoins are sampled again. Given the successful construction of an *s*- and *t*-quoin, if they have the value heads (tails) and tails (heads), respectively, the outcome of *g*-coin toss is heads (tails). If the outcome is the same each time, the protocol is repeated. From the successful sampling of the *g*_1_-coin, *f*_^_(*p*) can be constructed, as outlined earlier.

**Fig. 5:**
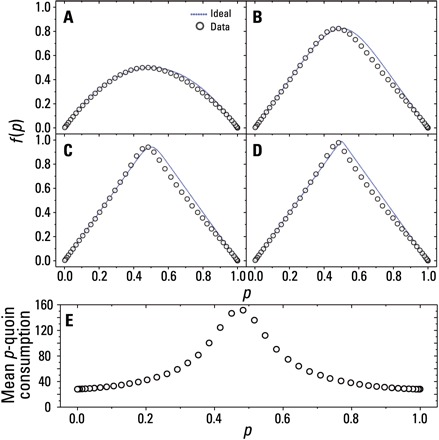
Experimental data for the single-qubit QBF. The function *f*_^_(*p*) = 2*p* is constructed using single-qubit measurements of *p*-quoins for (**A**) *k*_max_ = 1, (**B**) *k*_max_ = 10, (**C**) *k*_max_ = 100, and (**D**) *k*_max_ = 2000. The dotted blue lines are the ideal theoretical functions. Error bars were too small to be included (see Materials and Methods). (**E**) Mean *p*-quoin consumption for *k*_max_ = 82.

The experimental configuration is shown in Fig. 4B. Using the same photon-pair source as before, one photon is used as a herald, while the other is sent to an arrangement of MHWP, HWP, and a PBS. Again, the MHWP sets the bias *p*, while the HWP set to OA (OA + 22.5) enables *Z*-basis (*X*-basis) measurements to be performed for each *p*. Time-tags are recorded for each measurement basis independently, and the construction of *f*_^_(*p*) then follows by sampling from the two datasets. Figure 5 (A to D) shows experimental data (circles) taken for *k*_max_ ∈ {1, 10, 100, 2000}. The data show excellent agreement with theory under ideal conditions, albeit with a slight skew in the data which we attribute to mechanical drift in the fiber coupling. As one might expect, single-qubit measurements, which do not rely on nonclassical interference or multi-qubit coherence, can be performed with higher fidelity than joint measurements on two qubits. As such, in the case of *k*_max_ = 2000, we obtain *f*_^_(0.5) = 0.977 ± 0.006.

Of particular interest is a comparison of resource consumption between the two QBFs we have presented. For a fair comparison with the two-qubit QBF, we choose to restrict the series expansion of the single-qubit QBF to *k* = 82, which results in *f*_^_(0.5) = 0.935 ± 0.006. Figure 5E shows the mean *p*-quoin consumption for each *f*_^_(*p*)-coin. Averaging overall *p*, we require ≈ 52 quoins to construct *f*_^_(*p*) = 2*p* when using the quantum coherence and single-qubit measurements of *p*-quoins, which is approximately a fivefold increase in resources over the two-qubit case.

### Quantum advantage

Owing to small experimental imperfections, we are unable to exactly achieve *f*_^_(0.5) = 1 in our construction of *f*_^_(*p*) = 2*p*; however, this does provide an avenue for comparing the QBF to the CBF. We can frame the situation as a refereed game played between two parties, the quantum player who has a QBF and a classical player who has a CBF. The referee prepares *p*-quoins and sends them to the quantum player who is tasked with constructing, or approximating, *f*_^_(*p*) = 2*p* as best as they can. The quantum player can request a large, albeit finite, number of quoins. Their results—the quantum-obtained approximation to *f*_^_(*p*), for a range of *p* values—are sent to the classical player who must reproduce it using fewer resources. In the game, the quantum player achieves *f*_^_(0.5) = 1 − ϵ. The classical player’s strategy is as follows. First, they perform a least-squares fit of the data, for all *p*, using a positively weighted sum of Bernstein polynomials

$$\tilde{f}(p)=D\sum_{j=0}^{N}\frac{A_{j}}{D}(\begin{array}{c} N\\ j \end{array})p^{j}{\left(1-p\right)}^{N-j}=D.H(p)$$ (4)

where $D=\sum_{j=0}^{N}A_{j}$ and *D*. *H*(*p*) ≤ 1 − ϵ. The parameters *A*_*j*_ ≥ 0 are fitting parameters (see the Supplementary Materials) and ϵ = 1 − *f*_^_(0.5). As with the previously mentioned examples, polynomials of the form *p*^*j*^(1 − *p*)^*N* − *j*^ require *N* coins for exact sampling (*14*). This approach takes into consideration the nuances of the experimental data, which deviate from the ideal truncated function shown in Fig. 1B. It then follows from (*14*) that the mean coin consumption is

$$\bar{N_{c}}\sim \frac{9.5DN}{\epsilon }$$ (5)

To determine the optimal *N*, the classical player performs an optimization routine where the R-squared value is maximized for a range of *N*. For the data in Fig. 3D, ϵ = 0.065, *N* = 27, and *D* = 14.17 (see the Supplementary Materials), resulting in $\bar{N_{c}}\sim 56126$ coins, on average, which is three orders of magnitude greater than the quantum player, who thus wins the game. To the best of our knowledge, this is the optimum strategy that the classical player can use.

Last, we remark on how the resource consumption scales with ϵ. From (*14*), it was shown that the classical coin consumption for the truncated function shown in Fig. 1B is given by $\bar{N_{c}}\sim 19\epsilon^{-1}$. Taking into consideration the two-qubit QBF, we calculate the mean *p*-quoin consumption for a range of ϵ. The two-qubit QBF presented here shows an improvement where the mean quoin consumption scales as$\bar{N_{q}}\sim 2\epsilon^{-0.5}$, which is in broad agreement with the scaling derived from the experimental data, $\bar{N_{q}}\sim 3\epsilon^{-0.4}$. As expected, this further supports the notion of a quantum advantage in resource consumption over the best-known classical algorithm.

## DISCUSSION

The Bernoulli factory offers a fresh perspective from which information processing can be enhanced by quantum physics. Specifically, we have experimentally demonstrated a quantum advantage in the processing of randomness in a QBF under two different scenarios. Our work confirms that quantum coherence can provide a large reduction (three orders of magnitude) in resources over the CBF and that quantum entanglement provides a further fivefold reduction. An interesting but large and challenging theoretical extension would be to compare the resources for a CBF and a QBF to construct an approximation, *j*(*p*), to a general function *f*(*p*), to a certain degree of accuracy given some suitable measure between functions.

In addition, while our implementation uses bipartite entanglement, an interesting question is: How does this advantage scale when considering multipartite entangled systems? The QBF described here takes iid quoins as its input and outputs a coin. Lifting these restrictions, allowing quoins to be outputted rather than just coins is expected to give rise to other classes of factories and constructible functions (*20*, *21*).

In the study of quantum modeling protocols, the encoding of the input information with quantum states demonstrating coherence has also shown the potential to yield resource advantages in terms of memory (*22*–*25*). In particular, the QBF has recently drawn comparisons to the quantum transducer (*23*), which is a model of an input-output process requiring a lesser amount of past knowledge and complexity compared to its classical counterpart to simulate the future state of the system. Further investigation is required to determine whether the QBF can offer additional insight into the study of processes that have a causal dependence.

## MATERIALS AND METHODS

### Data processing

For each setting of *p*, ~ 3 × 10^6^ time tags were recorded. Since two photons are always created at the same time during the down-conversion process, the data were filtered, such that only tags occurring within a window of 6.25 ns remained. This process eliminates most of the spurious events due to ambient light or detector dark counts. The data were traversed, and a tally was kept of the number of coincidence detections corresponding to a heads outcome for each of the *g*_*k*_(*p*) ≡ *g*^*k*^_1_(*p*) coins as well as the number of tails outcomes. *g*_*k*_(*p*) was then calculated as #heads_*k*_/(#heads_*k*_ + #tails). We also note that a tally was kept of the total number of *p*-quoins required to produce an *f*_^_(*p*) = 2*p* coin, which also includes cases where coincident detections correspond to neither a head nor a tail *g*_1_(*p*)-coin. These events may occur due to imperfect extinction of the polarization optics or the finite polarization dependence of the nonpolarizing optics. This total is then weighted by *q*_*k*_, allowing the mean *p*-quoin consumption to be calculated.

Poissonian uncertainties arose because we counted a large number of *g*_*k*_-coins within a fixed data collection time window. Errors quoted throughout the main text were calculated, assuming Poissonian counting statistics of the coincidence detections, which are integrated to give #heads_*k*_ and #tails. Errors in *f*_^_(*p*) typically varied between ±2 × 10^− 4^ and ±3 × 10^− 3^.

## Supplementary Material

http://advances.sciencemag.org/cgi/content/full/5/1/eaau6668/DC1

## SUPPLEMENTARY MATERIALS

Supplementary material for this article is available at http://advances.sciencemag.org/cgi/content/full/5/1/eaau6668/DC1 Section S1. Constructing *g*_1_(*p*) in the single-qubit QBF Section S2. Bernstein polynomial fit of the data Fig. S1. Least-squares fit of *f*_^_(*p*) = 2*p*.
