# Supplementary material for: An experimental quantum Bernoulli factory
Source: Sci Adv. 2019 Jan 25;5(1):eaau6668. doi: 10.1126/sciadv.aau6668 (PMC6357723; doi:10.1126/sciadv.aau6668)
Supplement: http://advances.sciencemag.org/cgi/content/full/5/1/eaau6668/DC1 [file supp_5_1_eaau6668__index.html]

Science Advances | Science Advances

## Supplementary Materials

**This PDF file includes:**

- Section S1. Constructing *g*1(*p*) in the single-qubit QBF
- Section S2. Bernstein polynomial fit of the data
- Fig. S1. Least-squares fit of *f*^(*p*) = 2*p*.

Download PDF

**Files in this Data Supplement:**

- Adobe PDF - aau6668\_SM.pdf
