# Supplementary material for: An experimental quantum Bernoulli factory
Source: Sci Adv. 2019 Jan 25;5(1):eaau6668. doi: 10.1126/sciadv.aau6668 (PMC6357723; doi:10.1126/sciadv.aau6668)
Supplement: http://advances.sciencemag.org/cgi/content/full/5/1/eaau6668/DC1 [file aau6668_SM.pdf]

Science Advances
AAAS

advances.sciencemag.org/cgi/content/full/5/1/eaau6668/DC1

Supplementary Materials for

An experimental quantum Bernoulli factory

Raj B. Patel*, Terry Rudolph, Geoff J. Pryde*

*Corresponding author. Email: r.patel@griffith.edu.au (R.B.P.); g.pryde@griffith.edu.au (G.J.P.)

Published 25 January 2019, *Sci. Adv.* **5**, eaau6668 (2019)
DOI: 10.1126/sciadv.aau6668

This PDF file includes:

Section S1. Constructing $g_1(p)$ in the single-qubit QBF
Section S2. Bernstein polynomial fit of the data
Fig. S1. Least-squares fit of $f_\wedge(p) = 2p$.

Section S1. Constructing $g_1(p)$ in the single-qubit QBF

Following the treatment in ref. (19), here we detail the construction of the $g_1(p)$-quoin in the single-qubit QBF, as illustrated in Fig. 4A in the main text. First, we begin with two p-quoins, then second of can be measured in the X-basis produces a q-quoin with an outcome

$$\mathbb{P}_q(\text{Heads}) = 1 + 2\sqrt{p(1-p)} \tag{S1}$$

The p-quoin is tossed twice (upper branch) to generate a virtual m-quoin. Two different (identical) outcomes leads to a toss of the m-quoin with a value of heads (tails) with probability

$$\begin{aligned}\mathbb{P}_m(\text{Heads}) &= \mathbb{P}_p(\text{Heads})\mathbb{P}_p(\text{Tails}) \\ &+ \mathbb{P}_p(\text{Tails})\mathbb{P}_p(\text{Heads}) \\ &= 2p(1-p)\end{aligned} \tag{S2}$$

Similarly an n-quoin from two tosses of a q-quoin in the same manner giving

$$\begin{aligned}\mathbb{P}_n(\text{Heads}) &= \mathbb{P}_q(\text{Heads})\mathbb{P}_q(\text{Tails}) \\ &+ \mathbb{P}_q(\text{Tails})\mathbb{P}_q(\text{Heads}) \\ &= 1/2 - 2p(1-p)\end{aligned} \tag{S3}$$

The next step is to toss the m-quoin (n-quoin) twice if the first toss results in tails a we produce an s-quoin (t-quoin), with a tails outcome. If however, the first toss gives heads and the second gives tails then heads is outputted

$$\begin{aligned}
\mathbb{P}_s(\text{Heads}) &= \mathbb{P}_m(\text{Heads})\mathbb{P}_m(\text{Tails}) \\
&+ \mathbb{P}_m(\text{Heads})(1-\mathbb{P}_m(\text{Tails})\mathbb{P}_s(\text{Heads}) \\
\Rightarrow \mathbb{P}_s(\text{Heads}) &= \frac{m}{(1+m)}, \text{and} \qquad \text{(S4)}
\end{aligned}$$

$$\mathbb{P}_t(\text{Heads}) = \frac{n}{(1+n)} \qquad \text{(S5)}$$

Otherwise, the protocol is repeated. An s and t-quoin are tossed, if the result is heads (tails) and tails (heads), respectively, the outcome of g-quoin toss is heads (tails) with probability

$$\begin{aligned}
g_1(p) &\equiv \mathbb{P}_g(\text{Heads}) = \mathbb{P}_s(\text{Heads})\mathbb{P}_t(\text{Tails}) \\
&+ [1-\mathbb{P}_s(\text{Heads})\mathbb{P}_t(\text{Tails}) \\
&- \mathbb{P}_s(\text{Tails})\mathbb{P}_t(\text{Heads})]\mathbb{P}_g(\text{Heads}) \\
&= 4p(1-p) \qquad \text{(S6)}
\end{aligned}$$

else, the protocol is repeated.

Section S2. Bernstein polynomial fit of the data

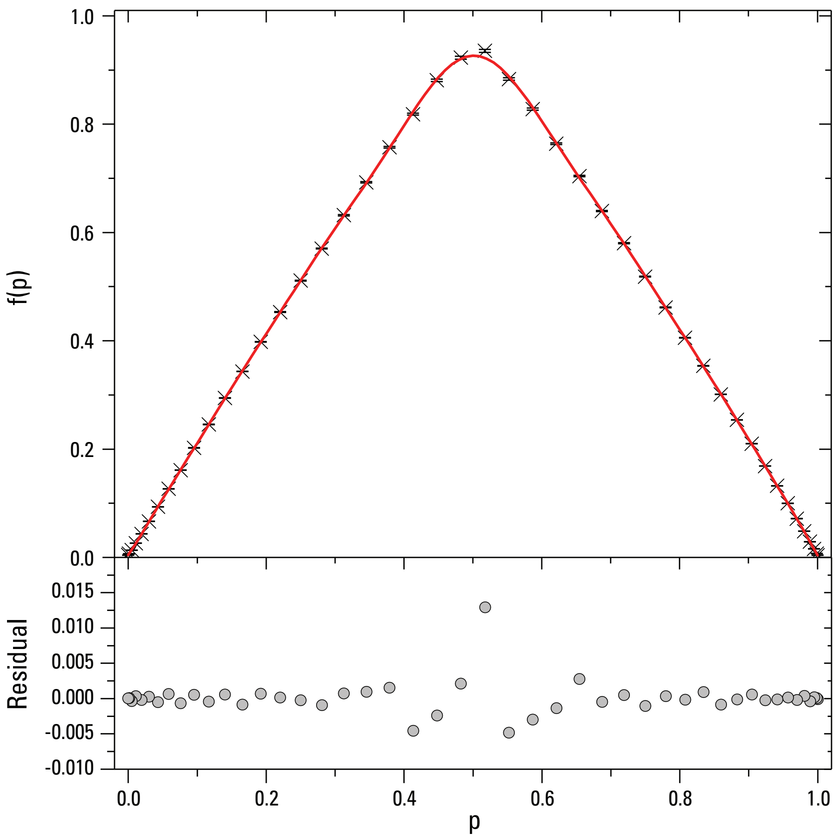

Fig. S1. Least-squares fit of $f_\wedge(p) = 2p$. For the $k = 2000$ of the two-qubit QBF, the experimental data was fitted using a sum of Bernstein polynomials given by Eq. 4 in the main text. The fit presented here for order $N = 27$ (and corresponding R-squared value of 0.999992) was determined by maximising the R-squared value for a range of N. The fit was weighted by the error bars shown, which were calculated assuming Poissonian statistics.
